# Is molecular evolution faster in the tropics?

**DOI:** 10.1038/s41437-018-0141-7

**Published:** 2018-09-10

**Authors:** Matthew G. Orton, Jacqueline A. May, Winfield Ly, David J. Lee, Sarah J. Adamowicz

**Affiliations:** 1 Department of Integrative Biology & Biodiversity Institute of Ontario, University of Guelph, 50 Stone Road East, Guelph, ON N1G 2W1 Canada; 2 School of Biological Sciences and Applied Chemistry, Seneca College, 1750 Finch Ave E, North York, ON M2J 2X5 Canada

**Keywords:** Molecular evolution, Evolutionary biology

## Abstract

The evolutionary speed hypothesis (ESH) suggests that molecular evolutionary rates are higher among species inhabiting warmer environments. Previously, the ESH has been investigated using small numbers of latitudinally-separated sister lineages; in animals, these studies typically focused on subsets of Chordata and yielded mixed support for the ESH. This study analyzed public DNA barcode sequences from the cytochrome *c* oxidase subunit I (COI) gene for six of the largest animal phyla (Arthropoda, Chordata, Mollusca, Annelida, Echinodermata, and Cnidaria) and paired latitudinally-separated taxa together informatically. Of 8037 lineage pairs, just over half (51.6%) displayed a higher molecular rate in the lineage inhabiting latitudes closer to the equator, while the remainder (48.4%) displayed a higher rate in the higher-latitude lineage. To date, this study represents the most comprehensive analysis of latitude-related molecular rate differences across animals. While a statistically-significant pattern was detected from our large sample size, our findings suggest that the EHS may not serve as a strong universal mechanism underlying the latitudinal diversity gradient and that COI molecular clocks may generally be applied across latitudes. This study also highlights the merits of using automation to analyze large DNA barcode datasets.

## Introduction

The latitudinal diversity gradient is one of the most striking and general features of biodiversity (Hillebrand 2004). While numerous ecological and evolutionary mechanisms have been proposed to explain this pattern (reviews by Mittelbach et al. 2007; Dowle et al. 2013; Gillman and Wright 2014; Fine 2015; Schluter 2016), the latitudinal diversity gradient remains an active and important area of enquiry in biodiversity science. According to the “evolutionary speed” hypothesis (ESH), the rate of evolution is higher in the tropics, likely due to shorter generation times, higher mutation rates, and/or a faster rate of selection (Rohde 1992). The rate of molecular evolution has been correlated with the rate of net diversification across a variety of taxonomic groups (Barraclough and Savolainen 2001; Davies et al. 2004; Lanfear et al. 2010; Bromham et al. 2015). Therefore, evolutionary speed could provide a general mechanism underlying the latitudinal diversity gradient across many taxonomic groups.

Despite the broad appeal and apparent explanatory power of this mechanism, there remains considerable uncertainty about whether rates of molecular evolution tend to be higher in the tropics. Findings have varied dramatically across different taxonomic groups and across studies. Significantly higher rates of molecular evolution have been detected at lower latitudes for diverse taxa, including mammals (Gillman et al. 2009), birds (Gillman et al. 2012), amphibians (Wright et al. 2010), aquatic turtles (Lourenço et al. 2013), marine fish (Wright et al. 2011), angiosperms (Davies et al. 2004; Wright et al. 2006; Gillman et al. 2010), and Foraminifera (Allen et al. 2006) (differences in elevation or water depth were considered together with latitude in some studies). By contrast, latitude was not significantly associated with branch lengths in lizards and snakes (Rolland et al. 2016), terrestrial turtles (Lourenço et al. 2013), water beetles (Fujisawa et al. 2015), or birds (Bromham and Cardillo 2003).

The wide variation in effect sizes among these studies may be partially explained by real differences in the strength of the latitude/rates correlation among taxa. In particular, ectotherms are expected to display a stronger trend than endotherms on theoretical grounds (Allen et al. 2006), as they experience variation in body temperature with environmental temperature; additional taxon-specific biological traits, environmental factors, and evolutionary processes may also contribute to the different findings. However, a large component of the variability may also be attributed to methodological differences. Diverse ecological, geographical, and phylogenetic inclusion criteria have been applied; some studies used only very closely-related sisters, for which relative rate estimation can be unreliable (Welch and Waxman 2008), while others included more phylogenetically-distant lineages, which may differ in many biological traits and habitat features (e.g. discussed in Wright et al. (2009)). Sample size has also varied considerably, with most studies analyzing a modest sample size (e.g. dozens of pairs), which may introduce noise into estimates of effect sizes. Additionally, sister-pair, whole-tree, and non-phylogenetic methodologies have all been employed, together with different genetic regions and a wide variety of methods for estimating branch lengths and branch length ratios between sister lineages. Methodological choices have been the topic of vigorous discussion (Weir and Schluter 2011; Gillman et al. 2011), and the conclusions drawn about variability in molecular evolutionary rates across latitudes influence the interpretation of general findings in macroevolution and macroecology (Weir and Schluter 2007, 2011; Gillman et al. 2009, 2011).

In this study, we use a large dataset of mitochondrial cytochrome *c* oxidase subunit I (COI) DNA sequences to test whether rates of molecular evolution are generally higher at lower latitudes in animals. By analyzing a dataset including eight thousand pairs of latitudinally-separated lineages spanning six animal phyla, and by using consistent analysis methods across taxa, we present the most comprehensive test to date of the generality of the molecular evolutionary speed hypothesis.

## Materials and Methods

### Filtering and aligning DNA sequence data sets

From Jan. 23-Mar. 17, 2017, we used the API of the Barcode of Life Data Systems (BOLD; Ratnasingham and Hebert 2007) to download all public specimen records for six animal phyla (SI: Parsing of BOLD datasets and Table 1). The records were next filtered to retain those with a Barcode Index Number (BIN; Ratnasingham and Hebert 2013) identifier, latitude (lat) and longitude (long), and a COI-5P barcode sequence. Sequences with internal gap or N content exceeding 1% of the total sequence length were eliminated. To facilitate alignment, sequences less than 640 base pairs (bp) and >1000 bp in length, not including gap characters, were also excluded. Sequences considered unlikely to be biologically relevant were removed from the analysis (SI: Sequence Removal Criteria). A single representative sequence (“centroid”) was selected from each BIN for further analysis. For each BIN, a DNA multiple sequence alignment was first performed. We performed all alignments using the muscle algorithm (Edgar 2004) from the R package muscle version 3.18.0 (available from http://bioconductor.org/packages/muscle/); details on alignment settings are available in SI: Alignment Settings. A pairwise distance matrix was then generated for each BIN using the TN93 (Tamura and Nei 1993) model of nucleotide substitution implemented in the R package Ape version 4.1 (Paradis et al. 2004). Details on choice of nucleotide substitution model are available in SI: Choice of Nucleotide Substitution Model. The centroid was defined as the sequence displaying the smallest average pairwise distance to all other sequences in the BIN.

**Table 1:** Summary of signed branch length ratios between pairs of Barcode Index Numbers (BINs) inhabiting lower vs. higher latitudes

Phylum class order	Number of Pairs			*P*-value (binomial test)	Median signed branch length ratio	P-value (Wilcoxon test of signed ratio)	Median, mean low/high latitude branch length ratio
	Total *N*	Positive (longer branch length in BIN closer to tropics)	Negative (longer branch length in BIN closer to poles)				
**Annelida**	**65**	**28 (43.1%)**	**37**	**0.321**	**−1.022**	**0.574**	**0.951, 1.028**
**Arthropoda**	**7130**	**3665 (51.4%)**	**3465**	0.018*	**1.001**	0.034*	**1.001, 1.039**
Arachnida	366	193 (52.7%)	173		1.004		1.002, 1.058
Collembola	63	32 (50.7%)	31		1.002		1.004, 1.109
Insecta***	6590	3389 (51.4%)	3201		1.001		1.001, 1.038
Coleoptera	844	421 (49.9%)	423		**−**1.000		1.000, 1.031
Diptera	1552	828 (53.1%)	724		1.002		1.002, 1.076
Hymenoptera	1011	531 (52.2%)	480		1.008		1.008, 1.069
Lepidoptera	2490	1255 (50.4%)	1235		1.004		1.006, 1.032
Malacostraca	111	51 (45.9%)	60		**−**1.006		0.994, 1.051
Chordata***	**677**	**365 (53.9%)**	**312**	0.045*	**1.012**	0.006**	**1.010, 1.095**
Actinopterygii	435	245 (56.3%)	190		1.016		1.013, 1.122
Perciformes	54	36 (66.7%)	18		1.005		1.014, 1.118
Aves	200	106 (53%)	94		1.005		1.006, 1.068
**Cnidaria**	**18**	**7 (38.8%)**	**11**	**0.481**	**−1.019**	**0.495**	**0.955, 1.118**
**Echinodermata**	**56**	**37 (66.1%)**	**19**	0.022*	**1.119**	0.0003**	**1.124, 1.384**
**Mollusca**	**91**	**44 (48.4%)**	**47**	**0.834**	**−0.026**	**0.456**	**0.999, 1.013**
**Overall**	**8037**	**4146 (51.6%)**	**3891**	0.0046**	**1.001**	0.0029**	**1.001, 1.046**

Results for phyla are marked in bold. Results are shown for selected taxa at the order or class level; these pairs are also included in the results for higher taxonomic levels. Mean lower/higher latitude ratios are reported in the last column for comparison to prior studies, but we suggest that signed branch length ratios better capture biological trends.

^*^Indicates a significant raw *p*-value.

**Indicates a significant *p*-value following sequential Bonferroni correction.

***Not all groups are represented; see Table S3 in SI for remaining groups in Insecta and Chordata.

The five smaller phyla and the majority of Arthropoda were analyzed at the class level, using the taxonomic hierarchy on BOLD. Analyzing most datasets at higher taxonomic levels permitted the inclusion of records lacking lower-level taxonomy (e.g. marine larvae). However, Insecta was analyzed at a lower taxonomic level due to computational limitations related to estimating genetic distances using a multiple sequence alignment for each taxon. Insecta was subdivided into Coleoptera, Diptera, Hymenoptera, Lepidoptera, and the remainder of Insecta. Due to their size (>20 K BINs), Hymenoptera, Diptera, and Lepidoptera were further subdivided into the separate geographical regions of North America, South America, Eurasia + Africa, and Australasia (SI: Geographic Divisions) for preliminary analysis and then recombined for final analysis.

For each taxonomic group, a DNA multiple sequence alignment and pairwise distance matrix were generated using the centroid sequences. Sequences exhibiting >0.15 pairwise distance to all others within its dataset were removed from analysis, as our next step involved analyzing closely-related BINs only; this step also resulted in the removal of contaminations involving phylogenetically distant taxa. For each of the taxonomic groups, we then selected a reference sequence (SI: Criteria for Reference Sequence Selection), which was trimmed to a standard length of 620 bp, for inclusion while generating a final alignment. Aligned sequences were then trimmed by the start and end positions of the reference sequence to standardize sequence length for further analysis.

### Pairing related lineages that differ in latitude

Using the final trimmed alignment for each dataset, preliminary pairings of BINs were first established by using pairwise distances. We do not assume that a mitochondrial gene represents the consensus species tree for our study taxa; rather, using a gene-specific approach to generate pairs for gene-specific molecular rates analysis overcomes problems related to incomplete lineage sorting, which can result in a bias towards estimating higher rates in more diverse taxa (Mendes and Hahn 2016). BINs exhibiting between 0.02 and 0.15 genetic divergence were considered candidate pairs. A minimum of 0.02 was set to avoid very small ingroup distances, which can yield extreme or unreliably-estimated (Welch and Waxman 2008) rate ratios when there are minimal changes in one or both members since the last common ancestor. Additionally, by using this lower threshold, we expect that the majority of our pairs will consist of different biological and evolutionary species (Ratnasingham and Hebert 2013), thus avoiding mixing intraspecific and interspecific comparisons. Our selected upper threshold reflects patterns of COI sequence variability that have been studied across four of the animal phyla included in our study, which indicate signs of transitional saturation at ca. 0.17–0.18 divergence (Carr 2010; Luo et al. 2011, Loeza-Quintana 2017). After conducting the test recommended by Welch and Waxman (2008), we found that branch length differences did not scale with branch length over our chosen range of divergences (SI: Testing for Unreliably Estimated Rate Ratios).

Candidate pairs passing the divergence criteria were retained if they additionally exhibited a difference of at least 20 degrees in median absolute latitude. Latitudes were converted from decimal degree format to absolute values prior to taking the median for each BIN. Original co-ordinates were used for calculating the median for mapping. Pairs in which the latitudinal range of either BIN overlapped by more than 25% with the latitudinal range of the other BIN were omitted. If a BIN occurred in multiple pairings that met the latitude criterion, we retained the pairing with the smallest ingroup distance (see Fig. 1 in Gillman et al. 2009, for justification). For those BINs bearing a species-level identification, median latitude values from BOLD were validated against latitude data obtained from the Global Biodiversity Information Facility (https://www.gbif.org) (further details in SI: GBIF Validation and Table S2). The latitudinal information obtained from the BOLD records corresponds well with the GBIF data for most groups and particularly so for well-studied taxa. It should be noted, however, that the groups that exhibited lower correlation values between the two datasets (e.g. Annelida, Cnidaria, and Collembola) are understudied and often present more challenges in terms of identification and cryptic diversity; we therefore argue that the use of Molecular Operational Taxonomic Units such as BINs, as opposed to traditional Linnaean species labels, will likely improve the accuracy of the latitudinal information for these lesser-known groups.

**Fig. 1:**
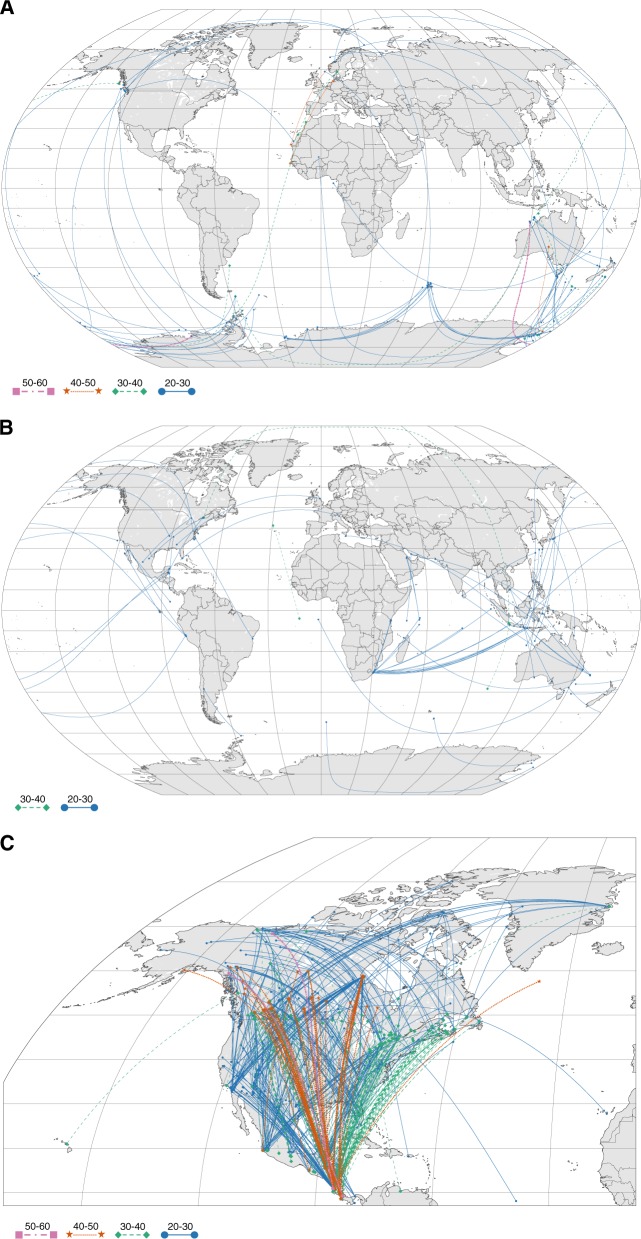
Sister lineages discovered for (**a**) Echinodermata (*n* = 56), (**b**) Perciformes (*n* = 54), and (**c**) Hymenoptera within North America (*n* = 517) separated by a minimum of 20 degrees in median absolute latitude and 0.02–0.15 sequence divergence, presented as examples of the total pairings analyzed in this study (*n* = 8037; see Fig. S7 for plots for all taxa). The point for each Barcode Index Number (BIN) included in a pair is plotted according to its median latitude and median longitude on Kavrayskiy VII map projections using the data visualization software plotly (https://plot.ly/). Pairings of lineages were color and symbol coded by the difference in median absolute latitude

For comparing relative rates of molecular evolution, it is advantageous to use a close relative for the outgroup, but it must be a valid outgroup that does not fall within the ingroup (Robinson et al. 1998). Therefore, each latitudinally-separated BIN pair was next assigned an outgroup, which was as closely related as possible yet also at least 1.3X more distant from each ingroup BIN than the ingroup distance. If an outgroup BIN was not available in a dataset that met the 1.3X divergence criterion for both lineages, then the pairing was omitted. Analysis was repeated using a 1.5X threshold for selected taxa; results were similar (SI: Choice of Outgroup Threshold).

### Signed branch length ratios for latitudinally-separated pairs

In addition to the ingroup distance, genetic distances to the outgroup were calculated for each member of a BIN pairing. In total, 53 pairings (0.65% of total pairings) were excluded where both members were equidistant from the outgroup. For each remaining pair, the three distances were used to estimate branch lengths for each ingroup member from their point of divergence, assuming an additive distance matrix for the three-taxon phylogeny. Branch lengths for each ingroup member (BL_a and BL_b) were calculated according to:

$$\begin{array}{l}{\rm{BL}}\_{\rm{a}} = \left( {{\rm{IG}}\_{\rm{ab/2}}} \right) + \left( {{\rm{OG}}\_{\rm{a}} - {\rm{OG}}\_{\rm{b/2}}} \right)\cr {\rm{BL}}\_{\rm{b}} = \left( {{\rm{IG}}\_{\rm{ab/2}}} \right) + \left( {{\rm{OG}}\_{\rm{b}} - {\rm{OG}}\_{\rm{a/2}}} \right)\end{array}$$

where IG_ab represents ingroup distance between ingroup members, OG_a represents distance to outgroup for ingroup member a, and OG_b represents distance to outgroup for ingroup member b. Distances for IG_ab, OG_a, and OG_b were calculated using the TN93 substitution model (Tamura and Nei 1993). Branch length ratio was then determined by dividing the larger of the two branch lengths (BL_a or BL_b) by the smaller of the two distance values. A positive or negative sign was then assigned to the branch length ratio for each pair based on direction, with positive signs used for pairs where the lower-latitude BIN exhibited a longer branch length. An alternative method for determining branch length ratios has been previously applied (e.g. Wright et al. 2011); thus, for comparison, we also calculated ratios by dividing the branch length with lower latitude over the branch length with higher latitude.

Phylogenetic pseudoreplication occurs when the same segments of branch length are included in multiple pairings, which would inflate the degrees of freedom for statistical testing. We investigated whether any ingroup sequence displayed a genetic distance to any sequence in any other ingroup pair in its dataset that was smaller than its own ingroup distance. In such instances, signed branch length ratios were averaged across the involved pairings to create a single data point prior to statistical testing (SI: Pseudoreplicate Determination and Averaging).

For each phylum and for selected large classes and orders, we tested whether there were significantly more than 50% positive signed branch length ratios using a binomial test. We additionally tested whether the median signed ratio differed from a null expectation of 0 using a Wilcoxon signed-rank test. Sequential Bonferroni correction (Holm 1979) was performed at the phylum level (Table 1).

### Linear regression analysis of phylogenetically independent contrasts

**Fig. 2:**
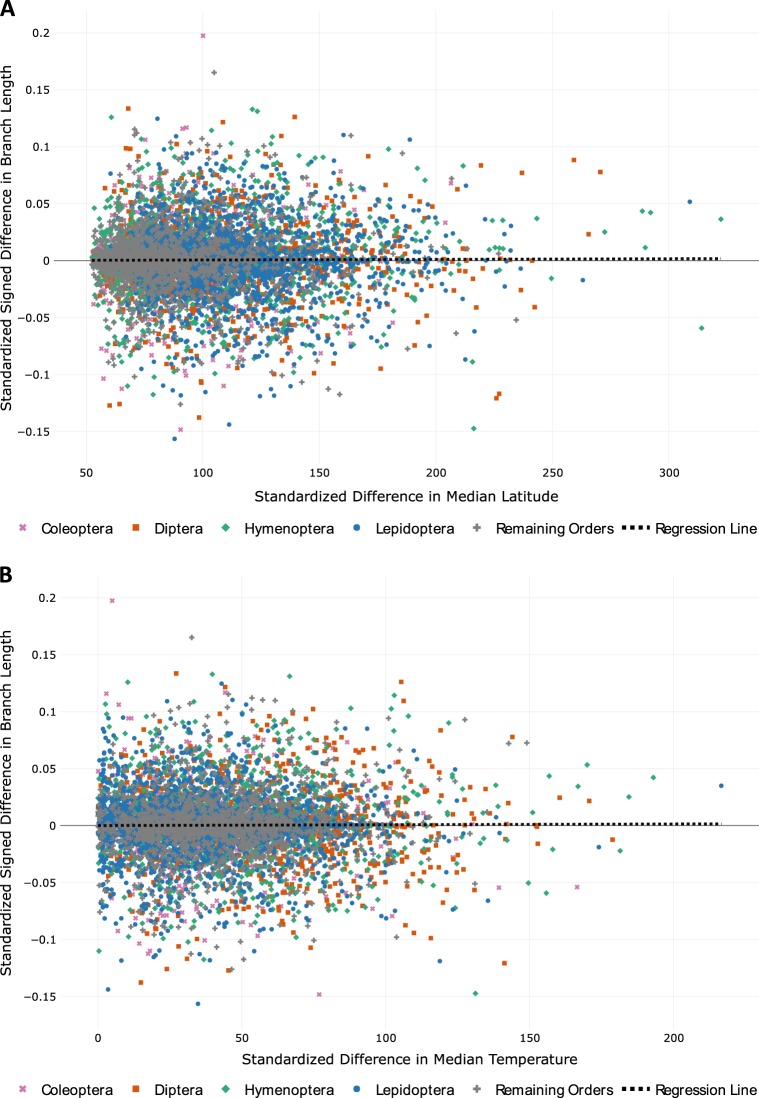
Linear regression analysis of standardized, phylogenetically independent contrasts (PICs) for all pairings of latitude-separated sister lineages belonging to Arthropoda (*n* = 7900), whereby each point represents one sister pair and the fitted regression line is forced through the origin. (**a**) PICs in median latitude vs. signed PICs in branch length (positive sign = larger branch length at lower latitude) (slope of regression = 5.36E-06). (**b**) PICs in median temperature vs. signed PICs in branch length (slope of regression = 6.73E-06). Each pairing of lineages is separated by a minimum of 20 degrees in median absolute latitude and 0.02–0.15 sequence divergence. Each pairing of lineages is color/symbol coded differently according to major insect order or gray/plus symbol for remaining orders within Arthropoda using the data visualization software plotly (https://plot.ly/)

Phylogenetically Independent Contrasts (PICs) were calculated, standardized for branch length, and subjected to regression analysis in R. Both latitude and temperature were considered as predictor variables, with branch length contrasts (i.e. standardized differences) as the response. Temperature data were obtained from the open source climate modeling tool WorldClim version 2 (http://worldclim.org/version2; Fick and Hijmans 2017; more details on these data available in SI: Parsing of Average Annual Global Temperature Data; Fig. 2). A linear regression model fitted through the origin was generated for each test (SI: Linear Regression Analysis of PICs and Table S5). A multiple regression of the PICs, including both temperature and latitude as predictors of branch length, was also performed for the Arthropoda data set (Table S5).

### Second and third codon position analysis

Linear regression analyses of PICs described above were performed for Arthropoda according to the 2nd and 3rd codon positions of the multiple DNA sequence alignments only. Divergences of pairings (TN93 substitution model) were recalculated according to the 2nd or 3rd codon position of the alignments, filtering out pairs with a divergence value of 0 at the second codon position; indeterminate (very high) divergence values between pairings were filtered out for the third codon position (SI: Codon-based analysis and Table S5).

### Whole tree-based analyses

Complementary tree-based analyses were also performed for selected taxonomic groups (Actinopterygii (ray-finned fishes), Aves (birds), and Papilionidae (swallowtail butterflies)) for comparison with the findings based upon the sister-pair approach. Maximum likelihood COI gene trees were constructed in RAxML (Stamatakis 2014) using a binary constraint tree from the literature, and the root-to-tip branch lengths were determined for each lineage. The method of phylogenetic generalized least squares (PGLS) (Grafen 1989) was employed, with the estimated branch lengths used as the response variable to test the effects of latitude on molecular evolutionary rate (for detail, see SI: Tree-based analyses).

### R code, platform, versioning and datasets

Taxonomic groups with fewer than/more than 10 K BINs were analyzed using the highly-commented R code publicly available through the following links:

https://github.com/m-orton/Evolutionary-Rates-Analysis-Pipeline/blob/master/EvolutionaryComparisonPipelineSmallTaxa.R

https://github.com/m-orton/Evolutionary-Rates-Analysis-Pipeline/blob/master/EvolutionaryComparisonPipelineLargeTaxa.R

Taxonomic groups with fewer than 10 K BINs were analyzed on the Elastic Compute Cloud instance r4.xlarge offered by Amazon Web Services, while those with more than 10 K BINs were run on the Elastic Compute Cloud instance r4.8xlarge (https://aws.amazon.com/ec2/). All taxonomic groups used a community Amazon Machine Image (AMI) created by Louis Aslett with R version 3.3.1 (https://www.r-project.org/) and R Studio version 0.99.903 (https://www.rstudio.com/) running on Ubuntu version 16.04.2 (64-bit).

## Results

Considering all six animal phyla, there was only a weak trend of higher rates of COI evolution in lineages inhabiting latitudes closer to the tropics. Of 8037 pairs of lineages exhibiting a contrast of at least 20 degrees in median absolute latitude and separated by 0.02–0.15 sequence divergence (Tamura-Nei model), 4146 (51.6%) displayed a higher rate of molecular evolution in the lower-latitude member, and 3891 had a higher rate in the higher-latitude lineage (binomial test *p*-value = 0.0046; Table 1). The median signed branch length ratio (larger/smaller branch length, with positive signs for pairs with higher rates at lower latitude) was 1.001, indicating a slight bias towards higher rates at lower latitudes (Wilcoxon test *p*-value = 0.0029). An alternative method for calculating branch length ratios (lower-latitude/higher-latitude branch length) yielded a similar directional pattern, with a median value of 1.001 and a mean value of 1.046.

While the overall results were dominated by the large sample size of Arthropoda, patterns were generally similar across taxa, including both endotherms and ectotherms (Table 1, Table S3). However, a few groups displayed a stronger association between molecular rates and latitude. For example, Echinodermata and Chordata exhibited higher rates of molecular evolution at lower latitudes significantly more often than expected by chance, including when using the Wilcoxon test following correction for multiple tests (Table 1). In addition to using the Tamura-Nei model for calculating genetic distances, the general time-reversible model (GTR + I + G) was also used for all phyla excluding Arthropoda and yielded a similar trend favouring higher rates at lower latitudes for both Chordata and Echinodermata when using the Wilcoxon test (SI: Choice of Nucleotide Substitution Model; Table S4).

Patterns were also similar to the overall trends when confining the analysis to those pairs in which one member inhabits the tropical zone (−23.437 to 23.437 latitude), with 3449 pairs (52.2%) exhibiting a higher rate at the lower latitude and 3152 (47.8%) with a higher rate at the higher latitude. In addition, trends were similar upon analyzing the subset of the data with the largest latitudinal differences. Of the 2304 pairs separated by ≥30° in median absolute latitude, 1193 (51.7%) of the pairs possessed positive signed ratios.

The results of the PICs for Arthropoda (Fig. 2 and Table S5) yielded little to no directional trend for both median latitude and median temperature with slope and R-squared values for each regression line being near 0, further supporting the results of the binomial and Wilcoxon tests in Table 1. When restricting PICs to either the 2nd and 3rd codon position of the DNA multiple alignments, this lack of directional trend remained largely unchanged (Table S5). Similarly, multiple regression including both temperature and latitude as predictors did not yield significant results (Table S6).

Finally, the results of the complementary tree-based analyses of selected taxa largely agree with the results of the sister pair pipeline (Table S7). Latitude appears to have either a non-significant effect (e.g. Aves, Papilionidae) or a slightly negative effect on molecular evolutionary rate (e.g. Actinopterygii), thus indicating a weak trend of higher rates at tropical latitudes in fish, in agreement with the sister-pair analysis for that taxon.

## Discussion

### Is molecular evolution faster in the tropics? Yes, but not by much

In this study, we set out to answer this question for animals, using a specific protein-coding region of the mitochondrial genome that has been widely used for taxonomic identification (Hebert et al. 2003), biodiversity research (e.g. Stahlhut et al. 2013), and molecular clock calibrations (Knowlton and Weigt 1998; Lessios 2008; Loeza-Quintana and Adamowicz 2018). In addition to using consistent analysis methods across six animal phyla, we used public DNA barcode data to generate the largest dataset to date of phylogenetically independent pairs of lineages differing in latitude (>8000 pairs), a ca. 50 to 150-fold larger sample size than in prior studies (Bromham and Cardillo 2003; Allen et al. 2006; Wright et al. 2011, 2006, 2010; Gillman et al. 2009, 2010, 2012; Lourenço et al. 2013; Fujisawa et al. 2015; Rolland et al. 2016). Despite several prior studies reporting a strong relationship between latitude and rates of molecular evolution, and others reporting no relationship, we found only a weak trend here. Generally, rates of COI evolution in animals were unrelated or only weakly related to latitude.

Nevertheless, we detected a statistically significant trend using our large sample, with just over half (51.6%) of the pairs exhibiting higher rates of molecular evolution closer to the tropics and stronger directional trends in specific taxonomic groups. Arthropods displayed a significant directional pattern, driven mainly by a slightly stronger trend and large sample sizes in the Arachnida and the insect orders Diptera and Hymenoptera. A significant trend (56% positive pairs) was also found within the bony fish (Actinopterygii), with the most noticeable trend observed in the large order Perciformes (67%), mirroring the overall directional pattern reported for marine fish by Wright et al. (2010), but with a smaller effect size. A marked directional trend was also found in the Echinodermata (66%), although the number of pairs for this phylum was small; this group would be worth further investigation after more comprehensive barcode coverage becomes available in public databases. In addition, when restricting the analysis to pairs differing by ≥30° in absolute median latitude, Echinodermata showed an even stronger directional trend of 82%. These taxon-specific latitudinal trends may be linked to biological traits that vary across groups. For example, within echinoderms, possible contributing mechanisms may include the longer generation times in polar regions as well as the commonality of a brooding mode of reproduction near the poles (Pearse et al. 2009), which can influence both rates and patterns of substitution (Foltz 2003). Multivariable methods (Bromham and Cardillo 2003; Fujisawa et al. 2015) could be further developed in the future to separate out the effects of latitude/temperature and traits upon molecular rates.

### Reconciling findings across studies: a matter of scale?

Our main finding of a near-even latitudinal pattern in COI rates contrasts markedly with the strong trends reported in several prior studies, which indicated that a large majority of pairs exhibited higher rates in the tropics or at higher temperatures or lower latitudes. Additionally, branch length ratios were large in some of these previous studies (e.g. 1.47 in Gillman et al. (2009), 1.61 in Wright et al. (2010), as contrasted with 1.05 overall here, using the same form of branch length ratio). Several factors may contribute to these discrepancies.

In particular, the phylogenetic scale of latitudinal comparisons may contribute to differing results. Generally, the studies reporting the largest effect sizes focused on very closely related taxa, which were paired such that ecological traits were as consistent as possible (Wright et al. 2006, 2010, 2011; Gillman et al. 2009). This approach has the benefit of focusing on effects relating to latitude or temperature differences as much as possible, while keeping other traits consistent. However, such pairings are somewhat subjective as to what qualifies a pair as being similar enough for inclusion. Moreover, the approach employed (Gillman et al. 2009) was criticized for the application of the stated inclusion/exclusion criteria, for model overfitting, and for the statistical analysis (Weir and Schluter 2011). Upon replying to these concerns, Gillman et al. (2011) reported a lower effect size than in the initial paper (1.28 vs. 1.47 for Cytochrome b for mammals inhabiting warmer vs. cooler environments), which was still higher than ratios reported here and in studies that considered more distantly related taxa (Bromham and Cardillo 2003) or that conducted whole-tree analysis for large taxonomic groups (Fujisawa et al. 2015; Rolland et al. 2016). In sum, these results suggest that rates may vary predictably with latitude (and/or elevation and depth) in very closely-paired taxa, for which unreliable rate ratio estimates are also more likely (Welch and Waxman 2008), but not more generally such that latitude would reliably predict rates across larger phylogenies.

Secondly, results may be impacted by the spatial scale of study as well as the way that environmental factors are measured. For example, Dugo-Cota et al. (2015) found no significant effect of latitude upon the rate of molecular evolution in glass frogs but a significant impact of temperature. Over the scale of their study, which was primarily confined to the tropics, latitude was not significantly correlated with temperature. The authors advocate using direct environmental data, rather than proxies such as latitude. In our study, all of our pairs differed by at least 20° in median absolute latitude, and 29% of pairs differed by more than 30°; as well, we analyzed temperature data in addition to latitude differences for these same pairs. Therefore, we would have expected to be able to detect an effect of temperature on rates if such a relationship were present. Our ability to compare with some of the prior research was limited due to modest COI barcode coverage for some vertebrate groups to date, due to historical preference for other markers for some vertebrate taxa (e.g. Cytochrome b). Nevertheless, our results agree with prior taxonomically and spatially broad studies that reported no latitudinal trend in branch lengths (Fujisawa et al. 2015; Rolland et al. 2016).

Despite methodological differences and varied findings among prior studies, we have found that our results converged between sister-pair and whole-tree analytical approaches for selected taxonomic groups we subjected to both analysis types (fish, birds, butterflies). For example, a PGLS regression analysis using 4658 species of fish and controlling for the number of nodes present along the root-to-tip distances revealed a significant negative correlation (*p* < 0.0001) between latitude and branch length, but again with a small effect size and low explanatory power (*R*^2^ = 0.11; Table S7), mirroring the sister-pair results. A subsequent multiple regression analysis performed by May (2017), who considered a total of 32 environmental and biological parametres, found that the significant effect of latitude disappeared when that variable was considered in a multivariable context. May (2017) discovered that life history traits, such as age at maturity, were more strongly related to branch lengths than environmental parametres. Therefore, latitudinal gradients in life history traits may contribute to explaining the weak latitudinal gradient in molecular rates that we detected here in the univariate analyses in some taxa, such as fish.

### Reconciling findings across studies: choice of gene region

An additional factor that may contribute to discrepant results among studies is the choice of genetic region(s). Due to its widespread adoption for DNA-based animal identification (Hebert et al. 2003) as well as biodiversity and applied research (Adamowicz 2015), the standardized COI barcode region has become the most-sequenced genetic region for many animal groups. There are currently >6 M DNA barcode records housed on the Barcode of Life Data Systems (BOLD, accessed June 25, 2018; Ratnasingham and Hebert 2007). Therefore, this marker is an ideal choice for performing a large-scale, single-marker study of evolutionary rates. Located at the core of cell respiration in an enzymatically active part of the cytochrome oxidase protein, the COI barcode region has been shown to be relatively conserved, likely via purifying selection, thus allowing for species-level identification, while also possessing variability at third codon positions as well as in specific regions of low functional constraint (Pentinsaari et al. 2016). These characteristics, combined with data availability, make the marker suitable for studies such as ours, which seeks to elucidate the factors that influence the rate of mitochondrial molecular evolution.

Other studies in animals that have investigated the relationship between molecular rates and latitude using mitochondrial markers have reported inconsistent findings to our own, whereby rates were found to be significantly higher at warmer latitudes. However, these studies have generally reported smaller sample sizes and have been mostly restricted to specific groups in Chordata including fishes and amphibians at the Cytochrome B (cyt b) gene as well as the 12S and 16S ribosomal RNA genes (Wright et al. 2010 and 2011), mammals at cyt b (Gillman et al. 2009), and turtles at COI, ND4, and cyt b (Lourenço et al. 2013). Birds, by contrast, have yielded inconsistent findings where significantly faster rates in the tropics were reported for cyt b in one study (Gillman et al. 2012), while there were no significant trends in cyt B or ND2 in another study (Bromham and Cardillo 2003). In arthropods, only one study has been performed which used the COI region in water beetles and reported insignificant results using phylogenetic methods and a large sample size of 5032 sequences (Fujisawa et al. 2015).

To date, few studies in animals have investigated the relationship between molecular rates and latitude at nuclear gene regions. Lourenço et al. (2013) reported statistically insignificant results at the RAG1, RAG2, and c‐mos gene regions in aquatic turtles. Insignificant results were also reported in scaled reptiles by Rolland et al. (2016) across 9 different nuclear genes and 3 mitochondrial genes using a larger sample size of 1651 species, analyzed through resampling sets of 51–141 species pairs differing in temperature.

Overall, it appears that choice of gene region (either mitochondrial or nuclear) can influence results in molecular rate studies in animals, with studies involving nuclear genes reporting no effect on molecular rates and studies involving mitochondrial genes generally reporting statistically significant trends at warmer latitudes but only when restricted to Chordata. However, the studies with the larger sample sizes, including Rolland et al. (2016) and Fujisawa et al. (2015), corroborate our findings based upon the COI barcode region that little to no trend in molecular rates is present when the molecular rate analyses are expanded to larger numbers of sequences.

### The evolutionary speed hypothesis and the latitudinal diversity gradient

Our primary finding of a weak latitudinal trend in molecular evolution rates was surprising. Several lines of evidence suggest that mutation rates are higher under higher energy expenditure, which can be facilitated by higher availability of environmental energy. While Lanfear et al. (2010) did not find a significant impact of metabolic rate upon molecular rates in a large comparative study, more recent research has indicated that active metabolic rate is a significant predictor of rates in poison frogs (Santos 2012), unlike basal metabolic rate, which may be decoupled from the rate of mitochondrial DNA evolution (Dowle et al. 2013). The availability of environmental energy relates to temperature as well as other conditions, particularly water availability (Goldie et al. 2010; Gillman and Wright 2014). Thus, evolutionary speed may be partially decoupled from latitude, as mediated through water and other resource availability, with variability across the Earth in these patterns. These considerations again point to the utility of further study using multivariable approaches.

In addition to the potential impact of energy availability upon mutation rates, latitudinal gradients in factors that influence the fixation rate should also be considered in the study of relative rates and molecular clocks. In general, the mutation rate is expected to predict the substitution rate for neutral mutations, regardless of population size, while selection is expected to eliminate or fix mutations with fitness consequences more readily in large populations. By contrast, nearly neutral, yet slightly deleterious mutations, which would be weeded out by natural selection in large populations, are expected to drift to fixation more readily when effective population size (*N*_e_) is small (Ohta 1992; Woolfit 2009). While abundance can be large in boreal regions, population extinction rates, and population size fluctuations may be lower in the tropics (Pyron and Wiens 2013), which may increase the long-term *N*_e_ of tropical species in contrast to temperate and polar regions. However, this trend remains to be explored across taxa. Large and growing datasets of standardized DNA sequences will open new avenues for studying gradients in evolutionary processes on a large spatial scale. Given that predictions arising from the nearly neutral theory have been borne out in comparative studies using mitochondrial DNA sequence data (e.g. Mitterboeck and Adamowicz 2013; Fujisawa et al. 2015; Mitterboeck et al. 2017), patterns of genetic variability may be further explored to test for variability in *N*_e_ across large spatial and taxonomic scales.

Evidence from selected taxa indeed suggests latitudinal structuring of traits correlated with *N*_e_. For example, Fujisawa et al. (2015) suggested that the weak (and nonsignificant) trend between rates and latitude that they observed in water beetles may have been an indirect effect of the latitudinal gradient in habitat availability/occupancy. In contrast to latitude, habitat (lentic vs. lotic) was a significant predictor of branch lengths, likely mediated by differences in *N*_e_ among the species occupying these two habitat types. Interestingly, Lourenço et al. (2013) discovered a habitat-by-latitude interaction in predicting rates in turtles. Whereas there was no difference in rates across latitudes in terrestrial turtles, aquatic turtles displayed a significant trend of higher rates at lower latitudes. As the aquatic designation was comprised of species inhabiting freshwater, marsh, and marine environments, an interesting avenue for further research would be to examine the latitudinal gradient in habitat occupancy in turtles and to test the correlation between rates and latitude/temperature within each of these habitat categories. These complex patterns suggest that future studies should target gradients in life history traits and *N*_e_, rather than latitude or temperature alone, and should employ multivariable analytical approaches in order to advance molecular rates research.

## Concluding remarks

As we found minimal variation in substitution rates across latitudes, we suggest that little to no correction for latitude may be required when using molecular clocks for COI to date evolutionary events for many animal taxa. However, further research is needed to test components of the ESH in more detail, including environmental correlates of variability in the mutation rate (as contrasted with substitution rate), investigating latitudinal gradients in habitat occupancy and *N*_e_, and characterizing spatial patterning in the speed of selection. Although COI did not show a substantial latitudinal pattern, even a modest increase in the rate of introduction of novel variants upon which selection can act may contribute to evolutionary speed in the tropics and may be one contributing factor towards the latitudinal diversity gradient. The potentially larger *N*_e_ in the tropics may permit the more effective action of positive selection at lower latitudes, a process which may influence other genes more strongly.

This research also highlights the utility and future promise of using large datasets of standardized sequences for evolutionary study, particularly when coupled with bioinformatics pipelines that enable automation and allow analyses to be repeated in the future in light of rapidly increasing data availability. This work also showcases the contribution that intensive biodiversity research at focal sites can make to global-scale studies in evolution and macroecology. Interestingly, targeted regional barcoding campaigns (e.g. Stahlhut et al. 2013; Wirta et al. 2016; Janzen and Hallwachs 2016) are apparent when viewing the latitudinally-separated pairs of Hymenoptera (Fig. 1C), even though the pairings were formed entirely informatically. Moreover, metabarcoding studies are increasingly generating multi-marker datasets of standardized molecular regions to reduce primer biases and improve taxon recovery (Cristescu 2014), which would allow research such as presented here to be expanded to markers beyond COI. Moreover, large data sets derived from metagenomics studies will create new opportunities for furthering our knowledge of the evolutionary origins, distribution, and future trends of biodiversity.

### Data Archiving

All DNA sequences analyzed in this work are publicly available through BOLD. The multiple sequence alignments with reference sequences for each taxonomic group studied and the results for the latitudinally-separated BIN pairs are now publicly available on Github though the following links:

https://github.com/m-orton/Evolutionary-Rates-Analysis-Pipeline/tree/master/SisterPairingDatasets

https://github.com/m-orton/Evolutionary-Rates-Analysis-Pipeline/tree/master/SupplementalDataSets

R code used to generate the results presented in this manuscript is publicly available through the Github links provided in the Materials and Methods section.

## Electronic supplementary material

Supplementary Material
