# Supplementary material for: Is molecular evolution faster in the tropics?
Source: Heredity (Edinb). 2018 Sep 10;122(5):513–24. doi: 10.1038/s41437-018-0141-7 (PMC6461915; doi:10.1038/s41437-018-0141-7)
Supplement: Supplementary file 1 — Supplementary Material [file 41437_2018_141_MOESM1_ESM.doc]

**Supplementary Information**

*Parsing of BOLD datasets*

Full data retrieval of both specimen and sequence data in TSV format was obtained using the BOLD API (<http://boldsystems.org/index.php/resources/api>) for six animal phyla (Table 1, main manuscript). Using the readr package in R (https://cran.r-project.org/web/packages/readr/index.html), downloaded TSVs were parsed and converted to dataframe format before use in the analyses.

*Sequence Removal Criteria*

Some sequences bearing a BIN identifier contained single bp insertions near the end of the sequence only, due to sequencing or editing error, especially in regions of homopolymer runs. These sequences were removed to generate biologically reasonable alignments that maintained homology. Additionally, sequences bearing a BIN identifier found to have insertions that were considered unlikely to be biologically relevant (e.g. insertions not in multiples of 3 nucleotides or insertions greater than 10 bp) were manually removed from the analysis.

*Alignment Settings*

Muscle alignments were run using the following parameters: diags set to True, gapopen set to -3000, and maxiters set to 3/2 for taxa smaller/larger than 10K BINs. The gap parameter setting was based upon preliminary analyses of Cnidaria, Echinodermata, and Mollusca, which each contained gapped regions. Alignments were performed starting with the default parameter values, followed by gradually increasing the gap penalty until nucleotide gaps were grouped together, reflecting entire amino acid insertions and deletions (indels). This gap opening penalty performed well across taxonomic groups in recovering biologically realistic alignments, which yielded conserved amino acid alignments upon translation.

*Choice of Nucleotide Substitution Model: Tamura-Nei (TN93) vs General Time Reversible (GTR)*

To explore the sensitivity of the results reported in Table 1 to model selection, we also used the GTR nucleotide substitution model with parameters I (proportion invariant sites) and G (gamma shape alpha) generated individually for each taxonomic group, excluding Arthropoda. G and I parameters for the GTR model were determined using the modelTest function in the R package phangorn (Schliep, 2011). Using these model parameters and the multiple sequence alignments for each group, pairwise distances between pairs of BINs were then calculated using the GTR+I+G distance settings in the phylogenetics program PAUP* V4.0 (Swofford 2002). Pairwise distance tables generated in PAUP were then imported back into R, where they were used in our bioinformatics pipeline to produce the results shown in Table S4. Overall, the results remain comparable to the TN93 model in terms of percentages of positive pairings and P-values for binomial and Wilcoxon tests. Annelida, Mollusca, and Cnidaria continue to show little to no trend, while a weak trend remains for Chordata, favouring higher molecular rates in the tropics. Echinodermata shows a weaker trend than the trend shown in Table 1 but does still show a trend when using the Wilcoxon test.

*Geographical Divisions for Diptera, Hymenoptera, and Lepidoptera*

Geographic regions were partly based on classical biogeographic regions while also ensuring each region contained a substantial latitudinal range and sample coverage. For North America (NA), we chose boundaries of -170 to -15 degrees longitude (long) and +7 to +90 latitude (lat), thus including Greenland and Mesoamerica; South America (SA) covered -135 to -30 lon and +7 to -90 lat; Australasia (AUS) was defined as -135 to +90 lon and 0 to -90 lat. Eurasia and Africa (EUR/AFR) included the remaining area not covered by these other regions for complete coverage of the earth. A summary of the signed branch length ratios for each geographic region for Diptera, Hymenoptera, and Lepidoptera is shown in Table S3.

*Criteria for Reference Sequence Selection*

Reference sequences were chosen that fell between the primer regions that amplify a specific portion of the 5’ end of the cytochrome c oxidase subunit I (COI-5P) gene (Folmer *et al.*, 1994), the animal barcode region (Hebert *et al.*, 2003). A reference BIN was first selected for each taxon using the following criteria: COI-5P sequences publicly available through BOLD, contains at least 10 sequences, meets the BIN metadata requirements, is associated with at least one specimen photograph, and has no taxonomic conflicts at the order level or above. The reference sequence was selected from each target BIN so as to be 658 bp long, have 2 trace files (chromatograms), and not contain any Ns or stop codons. In a few cases, we chose a reference sequence that was slightly longer than 658 bp, such as within Bivalvia, which had a reference sequence length of 661 bp due to an amino acid insertion in comparison with most animal barcodes. References sequences were trimmed symmetrically to a standard length of 620 bp and were used in the final alignment of each taxonomic group. Final alignments were trimmed to this standardized length and genetic region.

*Choice of Deletion Method: Complete Deletion vs Pairwise Deletion*

During the pairwise distance calculation between lineages, all groups used pairwise deletion of missing data with the exception of Mollusca, which used complete deletion due to some uncertainty of gap placement. For select taxa having many gaps (specifically Mollusca, Echinodermata, and Arachnida), we performed the analysis again basing the distance calculations on both pairwise deletion and complete deletion for comparison, using the same alignments for each group. Overall percentages of positive signed pairings varied somewhat between deletion methods, with positives for complete deletion of 48.3%, 59.6%, and 50.52% and positives for pairwise deletion of 42.6%, 66%, and 52.7% for Mollusca, Echinodermata, and Arachnida, respectively. Wilcoxon test p-values remained significant for Echinodermata and nonsignificant for Mollusca and Arachnida, suggesting that the choice of deletion method did not change the directional trends we observed in our results presented in the main manuscript (Table 1). As pairwise deletion would retain the most nucleotide data, we used that setting for most taxa.

*Testing for Unreliably-Estimated Rate Ratios*

Welch and Waxman (2008) outlined how estimates of substitution rates may violate standard assumptions of phylogenetically independent contrasts. Therefore, here we included non-parametric tests, including the binomial test, which considers direction alone, and Wilcoxon signed rank tests, which consider both direction and ranked magnitudes. For conducting the latter test, we explored whether our datasets contained evidence that branch length differences scale with branch length, as recommended by Welch and Waxman (2008). That is, are larger differences detected on longer branches? Therefore, for each phylum, we regressed the square-root transformed sum of branch lengths (our ingroup genetic distance) against the absolute value of the difference in estimated branch lengths for each ingroup pair. For all six phyla, we found a slope near zero (Table S1). For five phyla, the slope was not significantly different from zero. For Arthropoda, which contained an overwhelming sample size of pairs, the slope was significantly different from zero. However, the effect size (slope estimate) was very close to zero, as was the R^2^ value. For Arthropoda, a small number of extreme ratios was detected, but most values were between -2 and +2. Therefore, overall, we found that our approach of limiting our pairs to taxa with ≥2% divergence prevented there being a bias in branch length difference in relationship to genetic divergence. Therefore, we retained all pairs within the range of 2-15% divergence that also met the latitudinal difference criterion.

*GBIF validation*

To validate the latitude data obtained from the BOLD records, occurrence data were downloaded from the Global Biodiversity Information Facility (GBIF) on February 21^st^, 2018 via the R package rgbif (Chamberlain, 2017) for the taxonomic groups in Table 1. To match the BOLD latitudinal data with the corresponding GBIF latitudinal data, BINs containing sequences with species-level identifications were assigned a “species label”. The species label represented the majority species name for that BIN, if it was assigned to over 80% of the sequences present in the BIN. BINs without a clear species name consensus (less than 80% of the sequences had the majority species’ name) were not used in the analysis. The median values of the absolute latitude values for each species were then determined using the GBIF data. A correlation analysis was performed between the median latitude based upon BOLD vs. the median latitude from GBIF, for each taxonomic group (Table S2).

*Choice of Outgroup Threshold: 1.3x OG Divergence vs 1.5x OG Divergence*

To explore the impact of outgroup (OG) threshold choice, we also analyzed Chordata and Echinodermata using both the 1.3x OG divergence criterion and a 1.5x OG divergence criterion using the same alignments for each group. We found that numbers of positive signed ratios and median signed ratios changed slightly but that the directional trend stayed the same when changing the OG threshold. Wilcoxon test p-values remaining significant for both groups for both OG thresholds. Percentages of pairings with positive signed branch length ratios remained identical for Echinodermata and differed only by 1.6% for Chordata (53.9% for 1.3x and 55.5% for 1.5x).

*Pseudoreplicate Determination and Averaging*

A pairwise distance matrix was generated for latitudinally-separated lineages using the TN93 model (Tamura and Nei, 1993) with pairwise deletion of missing data. If a paired lineage was found to have a closer genetic distance to a lineage of another pairing, these lineage pairs would be grouped together as phylogenetic pseudoreplicates. Both signed and low/high branch length ratios of pairings with pseudoreplicate lineages were then averaged together before being used in the binomial and Wilcoxon tests. For signed ratios of pseudoreplicates differing in sign, we subtracted 1 from positive values and added 1 to negative values before averaging. After averaging, negative signed ratios had 1 subtracted and positive signed ratios had 1 added. This solution was implemented to address an issue where signed ratios of pseudoreplicates differing in sign were producing averages that were difficult to interpret biologically.

*Tree-based analyses*

Complementary tree-based analyses were performed for three taxonomic groups that are well represented on BOLD: ray-finned fishes (Actinopterygii), swallowtail butterflies (Papilionidae), and birds (Aves) (code adapted from <https://github.com/jmay29/phylo>). These analyses also followed the same filtering steps and centroid sequence selection and alignment process as the sister pair analytical pipeline. However, only those BINs with species-level information were included in the analysis, as this information was required to match with backbone phylogenies used in the tree-building process. “Species labels” were assigned to BINs containing sequences with species-level identifications. The species label represented the majority species name for that BIN, if it was assigned to over 80% of the sequences present in the BIN. BINs without a clear species name consensus (less than 80% of the sequences had the majority species’ name) were discarded. Median latitude values for each species were determined in the same manner as for the sister pair pipeline. Upon alignment of the centroid sequences, quality checks were performed to remove extremely “gappy” or divergent sequences (i.e. outliers or potentially contaminated sequences) from the dataset. For each taxonomic group, maximum likelihood COI gene trees were built in RAxML version 8.0 (Stamatakis, 2014) using the centroid sequence alignments. Backbone constraints based on multiple markers were obtained from the literature and used for each analysis (see Table S7). Functions from the R packages ape (Paradis et al., 2004), caper (Orme et al., 2013), and phytools (Revell, 2017) were used to prepare the molecular rate data and perform phylogenetic generalized least squares (PGLS) (Grafen, 1989). The branch lengths of the trees were estimated using all three codon positions and re-estimated again using only third codon positions. To quantify the rate of molecular evolution, the branch lengths (i.e. the mean number of substitutions per site) were summed up from root-to-tip for each species. The gene trees were then made ultrametric using the penalized likelihood method (Sanderson, 2002) prior to their incorporation into the PGLS model; a value of 177 was used as the smoothing parameter (lambda), which was the median of the optimized lambda values across data sets in Sanderson (2002). Branch length was specified as the response variable and median latitude as the explanatory variable (Table S7). To control for the node density effect, which can introduce bias when utilizing root-to-tip distances, the number of nodes from root-to-tip was also determined for each species and included as a control variable in each analysis. In general, the results of the PGLS analyses support the findings of the sister pair approach, as the effect of median latitude on molecular evolutionary rate appeared either non-significant (Aves, Papilionidae) or slightly negative (Actinopterygii) (Table S7).

*Parsing of Average Annual Global Temperature Data*

To perform an additional analysis considering temperature differences between pairings, average annual land-based global temperature data (5 km^2^ spatial resolution) from WorldClim version 2 (Fick, 2017) was imported into R using the R package raster (Hijmans, 2017) and then matched approximately (lat and lon values rounded to 1 decimal place) to the geographic coordinates of the latitude-separated BIN pairings discovered for the full Arthropoda dataset.

*Linear Regression Analysis of Phylogenetically Independent Contrasts*

Median temperature difference (using temperature data described above) and median latitude difference (using BOLD data) between each BIN pairing of Arthropoda were calculated, converted to an absolute value and standardized according to the square root of the sum of branch lengths of each pairing. The branch length differences of each species pairing were calculated, converted to an absolute value, standardized according to the square root of the sum of branch lengths, and signed (positive sign = larger branch length at lower latitude, negative sign = larger branch length at higher latitude). Phylogenetically Independent Contrasts (PICs) were performed for standardized median latitude difference vs. standardized signed branch length difference and for standardized median temperature difference vs. standardized signed branch length difference (can be seen plotted in panels A and B of Figure 2 in main manuscript). A linear regression line fitted through the origin was then generated for each test (details in Table S5). Overall, no significant directional trend was found across either of the PIC regressions, with the slope of each regression line being near 0 (5.36E-06 for latitude and 6.73E-06 for temperature**)** and R^2^ values also being close to 0 (2.59E-04 for latitude and 1.30E-05 for temperature).

*Codon-based analyses*

Linear regression analyses of PICs described above were also performed for the full Arthropoda dataset but according to the 2nd and 3rd codon positions of the multiple DNA alignments only. Genetic distances of pairings (TN93 substitution model) were recalculated according to the 2nd or 3rd codon position of the multiple DNA alignment, filtering out pairs with no divergence at the second codon position; indeterminate divergence values between pairings were filtered out of the analysis at the third codon position. Pairings found to have negative branch length values (instances where ingroup distance between lineages exceeded outgroup distances) were filtered out of the analysis. No significant directional trend was found at either codon position for either of the latitude or temperature PICs (Table S5).

Table S1: Results of fitting a linear model of the square-root transformed sum of branch lengths against the absolute value of the difference in estimated branch lengths for each ingroup pair from their point of divergence.

| Phylum | **Range of Signed Relative Branch Length Ratios** | **Slope** | **Multiple R-Squared** | **Adjusted R-Squared** | **P-Value** | **F-Statistic** | **DF^1^** |
| --- | --- | --- | --- | --- | --- | --- | --- |
| Annelida | -5.232 – 2.089 | 0.00197 | 0.00035 | -0.01435 | 0.8771 | 0.02412 | 68 |
|  |  |  |  |  |  |  |  |
| Arthropoda | -17.693 – 8.4265 | -0.01065 | 0.01244 | 0.01232 | 2.2e-16 | 104.3 | 8283 |
|  |  |  |  |  |  |  |  |
| Chordata | -5.838 – 6.0282 | -0.00749 | 0.00415 | 0.00271 | 0.0894 | 2.893 | 694 |
|  |  |  |  |  |  |  |  |
| Cnidaria | -1.756 – 5.253 | -0.00566 | 0.00560 | -0.04964 | 0.7537 | 0.1015 | 18 |
|  |  |  |  |  |  |  |  |
| Echinodermata | -1.960 – 5.552 | -0.01645 | 0.01611 | -0.00056 | 0.3296 | 0.9662 | 59 |
|  |  |  |  |  |  |  |  |
| Mollusca | -1.668 – 2.784 | -0.01016 | 0.00705 | -0.00188 | 0.3763 | 0.789 | 111 |

^1^ All detected pairs were included in this test for a scaling pattern, prior to checking for and combining phylogenetic pseudoreplicates.

Table S2: Results of correlations between median latitudinal data obtained from BOLD vs. GBIF.

| **Taxa** | **# of pairs^1^** | **# of BINs** | **BINs containing sequences with species-level information (%)** | **# BINs with species information found on GBIF (Total N for correlation)** | **Pearson Correlation Coefficient (r)** | **R-Squared** | **P-Value** |
| --- | --- | --- | --- | --- | --- | --- | --- |
| Annelida | 70 | 140 | 50.71 | 42 | 0.678 | 0.404 | 0.00000365* |
| Arachnida | 437 | 874 | 36.50 | 291 | 0.795 | 0.631 | < 2E-16* |
| Collembola | 80 | 160 | 19.38 | 23 | 0.361 | 0.0891 | 0.0903 |
| Coleoptera | 949 | 1898 | 38.41 | 617 | 0.824 | 0.678 | < 2E-16* |
| Diptera | 1690 | 3380 | 17.81 | 431 | 0.819 | 0.67 | < 2E-16* |
| Hymenoptera | 1186 | 2372 | 19.77 | 275 | 0.845 | 0.719 | < 2E-16* |
| Lepidoptera | 2907 | 5814 | 38.36 | 1344 | 0.858 | 0.736 | < 2E-16* |
| Malacostraca | 127 | 254 | 53.94 | 113 | 0.888 | 0.787 | < 2E-16* |
| Actinopterygii | 459 | 918 | 53.38 | 453 | 0.791 | 0.625 | < 2E-16* |
| Perciformes | 65 | 130 | 64.62 | 82 | 0.798 | 0.633 | < 2E-16* |
| Aves | 237 | 474 | 87.76 | 394 | 0.809 | 0.653 | < 2E-16* |
| Cnidaria | 20 | 40 | 52.50 | 14 | 0.544 | 0.238 | 0.0441* |
| Echinodermata | 61 | 122 | 51.64 | 53 | 0.842 | 0.703 | 2.85E-15* |
| Mollusca | 113 | 226 | 53.54 | 80 | 0.826 | 0.679 | < 2E-16* |

^1^The numbers of pairs refers to the original number of BIN pairings, prior to pseudoreplication averaging.

*Indicates a significant p-value.

Table S3: Summary of signed branch length ratios between pairs of BINs inhabiting lower vs. higher latitudes for the datasets of Elasmobranchii, Mammalia, regional datasets for Diptera, Hymenoptera, and Lepidoptera, and the remaining orders of Insecta not included in Table 1 in the main manuscript. Mean lower/higher latitude ratios are reported for comparison to prior studies, but we suggest that signed branch length ratios better capture biological trends. Regional datasets contain a different number of total pairs (*n*=1552, *n*=1086, *n*=2653) than the combined datasets (*n* =1590, *n*=1011*, n*=2490) for the insect orders Diptera, Hymenoptera, and Lepidoptera, respectively.

| Class  Order  Geographical  Region^1^ | **Number of Pairs** | | | **Median Signed Branch Length Ratio** | **Median, Mean**  **Low/High Latitude**  **Branch Length Ratio** |
| --- | --- | --- | --- | --- | --- |
|  | **Total N** | **Positive**  **(Longer Branch Length in BIN Closer to Tropics)** | **Negative**  **(Longer Branch Length in BIN Closer to Poles)** |  |  |
| Elasmobranchii | 15 | 7 (44.4%) | 8 | -1.015 | 1.100, 1.050 |
|  |  |  |  |  |  |
| Mammalia | 20 | 8 (40%) | 12 | -1.026 | 0.980, 1.004 |
|  |  |  |  |  |  |
| Insecta | 6590 | 3389 (51.4%) | 3201 | 1.001 | 1.001, 1.038 |
| Diptera | 1590 | 724 (53.3%) | 672 | 1.002 | 1.002, 1.076 |
| North America | 929 | 485 (52.2%) | 444 | 1.001 | 1.001, 1.030 |
| South America | 154 | 82 (53.2%) | 72 | 1.001 | 1.001, 1.017 |
| Eurasia+Africa | 419 | 236 (56.3%) | 183 | 1.005 | 1.005, 1.054 |
| Australasia | 50 | 25 (50%) | 25 | -0.0001 | 0.998, 1.038 |
|  |  |  |  |  |  |
| Hymenoptera | 1011 | 531 (52.2%) | 480 | 1.008 | 1.008, 1.069 |
| North America | 517 | 282 (54.5%) | 235 | 1.009 | 1.005, 1.064 |
| South America | 92 | 47 (51%) | 45 | 1.003 | 1.000, 1.044 |
| Eurasia+Africa | 419 | 219 (52.2%) | 200 | 1.009 | 1.004, 1.061 |
| Australasia | 58 | 28 (48.2%) | 30 | -1.007 | 1.008, 1.066 |
|  |  |  |  |  |  |
| Lepidoptera | 2490 | 1255 (50.4%) | 1235 | 1.004 | 1.006, 1.032 |
| North America | 753 | 368 (48.8%) | 385 | -1.001 | 0.999, 1.018 |
| South America | 502 | 262 (52.1%) | 240 | 1.002 | 1.002, 1.054 |
| Eurasia+Africa | 1042 | 522 (50%) | 520 | 1.000 | 1.000, 1.036 |
| Australasia | 356 | 187 (52.5%) | 169 | 1.001 | 1.002, 1.021 |
|  |  |  |  |  |  |
| Remaining Insect Orders^2^ | 811 | 419 (51.6%) | 392 | 1.002 | 1.003, 1.031 |

^1^Refer to SI for precise boundaries on geographical regions.

^2^Refers to all other orders within Insecta not including Coleoptera, Diptera, Hymenoptera, and Lepidoptera

Table S4: Summary of signed branch length ratios between pairs of BINs inhabiting lower vs. higher latitudes for Annelida, Chordata (subdivided by class), Cnidaria, Echinodermata, and Mollusca. In contrast to the results in Table 1 where pairwise distances are calculated using the TN93 model, pairwise distances between pairs of BINs were calculated using the GTR+G+I model.

| **Phylum**  Class | **Number of Pairs** | | | **P-Value (Binomial Test)** | **Median Signed Branch Length Ratio** | **P-Value (Wilcoxon Test of**  **Signed**  **Ratio)** | **Median, Mean**  **Low/High Latitude**  **Branch Length Ratio** |
| --- | --- | --- | --- | --- | --- | --- | --- |
|  | **Total N** | **Positive**  **(Longer Branch Length in BIN Closer to Tropics)** | **Negative**  **(Longer Branch Length in BIN Closer to Poles)** |  |  |  |  |
| **Annelida** | **87** | **40 (45.9%)** | **47** | **0.52** | **-1.006** | **0.465** | **0.999, 1.08** |
|  |  |  |  |  |  |  |  |
| **Chordata** | **583** | **322 (55.2%)** | **261** | **0.013*** | **1.019** | **0.008*** | **1.022, 1.135** |
| Actinopterygii | 369 | 211 (57.2%) | 158 |  | 1.045 |  | 1.048, 1.174 |
| Aves | 173 | 87 (50.3%) | 86 |  | 1.001 |  | 1.001, 1.052 |
| Elasmobranchii | 14 | 9 (64.3%) | 5 |  | 1.072 |  | 1.1, 1.152 |
| Mammalia | 27 | 15 (55.5%) | 12 |  | 1.015 |  | 1.015, 1.116 |
|  |  |  |  |  |  |  |  |
| **Cnidaria** | **16** | **9 (56.3%)** | **7** | **0.804** | **1.013** | **0.562** | **1.013, 1.401** |
|  |  |  |  |  |  |  |  |
| **Echinodermata** | **56** | **30 (53.6%)** | **26** | **0.689** | **1.045** | **0.039*** | **1.045, 1.441** |
|  |  |  |  |  |  |  |  |
| **Mollusca** | **90** | **44 (44%)** | **56** | **0.271** | **-1.031** | **0.057** | **0.976, 1.056** |

*Indicates a significant p-value.

Table S5: Linear regression analysis results of standardized, phylogenetically independent contrasts (PICs) for all pairings of latitude-separated sister lineages belonging to Arthropoda (total *n* = 7900, including all original pairs). Median latitude and median temperature differences between pairings were standardized according to branch lengths between each lineage of a pairing and converted to absolute values before being used in the regression. PICs were also performed for the second (*n* = 1365) and third (*n* = 1703) codon positions of Arthropoda. Each pairing of lineages is separated by a minimum of 20 degrees in median absolute latitude and 0.02-0.15 overall sequence divergence. For the second and third codon position analyses performed, sequence divergences of pairings were recalculated based on codon position, filtering out pairs with no divergence at the second position; indeterminate divergences between pairings were filtered out of the analysis at the third position.

| **Phylum**  Class  Order | **Codon**  **Positions** | **DF** | **Linear regression analysis: PICs in median latitude difference vs signed branch length difference of pairings** | | | | **Linear regression analysis: PICs in median temperature difference vs signed branch length difference of pairings** | | | |
| --- | --- | --- | --- | --- | --- | --- | --- | --- | --- | --- |
|  |  |  | **Slope** | **F-Statistic** | **R-Squared** | **P-Value** | **Slope** | **F-Statistic** | **R-Squared** | **P-Value** |
| **Arthropoda** | **1, 2, 3** | **7899** | **5.36E-06** | **3.046** | **2.59E-04** | **0.081** | **6.73E-06** | **1.103** | **1.30E-05** | **0.294** |
|  | **2** | **1364** | **3.34E-06** | **0.479** | **-3.81E-03** | **0.488** | **8.87E-06** | **0.867** | **-9.69E-05** | **0.352** |
|  | **3** | **1703** | **4.65E-05** | **0.792** | **-1.21E-03** | **0.373** | **1.00E-03** | **0.325** | **-3.96E-03** | **0.568** |
| Arachnida | 1, 2, 3 | 416 | 2.09E-05 | 1.701 | 1.68E-03 | 0.193 | 5.34E-05 | 3.750 | 6.55E-03 | 0.053 |
| Collembola | 1, 2, 3 | 79 | -2.18E-05 | 1.198 | 2.47E-03 | 0.277 | -4.85E-05 | 1.841 | 1.04E-02 | 0.179 |
| Insecta | 1, 2, 3 | 7358 | 5.47E-06 | 3.013 | 2.74E-04 | 0.083 | 5.63E-06 | 0.706 | -4.00E-05 | 0.401 |
| Coleoptera | 1, 2, 3 | 910 | -6.09E-06 | 0.344 | -7.21E-04 | 0.558 | -2.98E-05 | 1.902 | 9.89E-04 | 0.168 |
| Diptera | 1, 2, 3 | 1638 | 5.12E-06 | 0.677 | -1.97E-04 | 0.411 | 5.46E-06 | 0.230 | -4.70E-04 | 0.632 |
| Hymenoptera | 1, 2, 3 | 1140 | 2.95E-05 | 9.739 | 7.60E-03 | 0.002* | 4.46E-05 | 5.721 | 4.12E-03 | 0.017* |
| Lepidoptera | 1, 2, 3 | 2793 | -5.91E-07 | 0.017 | -3.52E-04 | 0.896 | -1.29E-05 | 1.129 | 1.04E-04 | 0.256 |
| Remaining Orders | 1, 2, 3 | 873 | 1.76E-06 | 0.029 | -1.11E-03 | 0.865 | 2.03E-05 | 0.910 | -1.03E-04 | 0.340 |
| Malacostraca | 1, 2, 3 | 43 | -1.14E-04 | 4.759 | 7.87E-02 | 0.035* | -2.12E-04 | 4.634 | 7.63E-02 | 0.037* |

*Indicates a significant p-value.

Table S6: Multiple regression results of phylogenetically independent contrasts (PICs) for all pairings of latitude-separated sister lineages belonging to Arthropoda (*n* = 7900). Analysis was performed on standardized median absolute temperature difference and average latitude vs standardized branch length difference between pairings. Each pairing of lineages is separated by a minimum of 20 degrees in median absolute latitude and 0.02-0.15 sequence divergence.

| **Phylum** | **Total N** | **DF** | **Model EstimatedR-Squared** | **Model Estimated**  **P-Value** | **F-Statistic** | **Model Coefficients** |
| --- | --- | --- | --- | --- | --- | --- |
| Arthropoda | 7900 | 7898 | 3.95E-05 | 0.3148 | 1.156 | 1.392E-05^1^, -1.556E-05^2^ |

^1^Standardized median temperature difference between pairings of lineages.

^2^Average latitude between pairings of lineages (signed and unstandardized).

Table S7: Results of PGLS tree-based analyses for select taxonomic groups that tested the effect of median latitude on root-to-tip branch length. Number of nodes was included as a control variable in each analysis.

| **Taxa** | **Backbone phylogeny used** | **DF** | **Codon Positions** | **Model Estimated R-Squared** | **Model Estimated P-Value** | **F-Statistic** | **Pagel’s** λ**^1^** | **Median Latitude** | | **Number of Nodes** | |
| --- | --- | --- | --- | --- | --- | --- | --- | --- | --- | --- | --- |
|  |  |  |  |  |  |  |  | **Slope** | **P-Value** | **Slope** | **P-Value** |
| Actinopterygii | Betancur-R et al. (2013) | 4658 | 1, 2, 3 | 0.11 | < 2.2E-16* | 260.9 | 0.97 | -0.00036 | 0.00088* | 0.0088 | < 2E-16* |
|  |  |  | 3 | 0.080 | < 2.2E-16* | 188.2 | 0.99 | -0.0012 | 6.613E-07* | 0.016 | < 2E-16* |
| Aves | Prum et al. (2015) | 1771 | 1, 2, 3 | 0.095 | < 2.2E-16* | 93.63 | 0.98 | 0.000015 | 0.69 | 0.0035 | < 2E-16* |
|  |  |  | 3 | 0.036 | < 2.09E-16* | 34.46 | 1.00 | 0.00017 | 0.47 | 0.010 | 4.44E-16* |
| Papilionidae | Nazari et al. (2007) | 101 | 1, 2, 3 | 0.13 | 0.00025* | 9.00 | 1.00 | -0.00024 | 0.29 | 0.0087 | 0.000065* |
|  |  |  | 3 | 0.090 | 0.0029* | 6.18 | 1.00 | -0.0010 | 0.64 | 0.071 | 0.00068* |

^1^Pagel’s λ is estimated through maximum likelihood and provides a value that best transforms the phylogenetic variance-covariance matrix of a trait to fit the observed data (Pagel, 1999).

*Indicates a significant p-value.

**References cited in supplementary file**

Betancur-R, R., Broughton, R.E., Wiley, E.O., Carpenter, K., López, J.A., Li, C., et al. (2013). The tree of life and a new classification of bony fishes. *PLoS Curr Tree of Life* **2013 Apr 18**: Edition 1.

Orme CDL, Freckleton R, Thomas G, Petzoldt T, Fritz S, Isaac N, Pearse W. (2013). Caper: comparative analyses of phylogenetic and evolution in R. R package version 0.5.2.

Chamberlain (2017). rgbif: Interface to the Global ‘Biodiversity’ Information Facility ‘API’. R package version 0.9.8.

Fick SE, Hijmans RJ (2017). WorldClim 2: new 1-km spatial resolution climate surfaces for global land areas. *Int J Climatol* **37**: 4302–4315.

Folmer O, Black M, Hoeh W, Lutz R, Vrijenhoek R (1994). DNA primers for amplification of mitochondrial cytochrome c oxidase subunit I from diverse metazoan invertebrates. *Mol Mar Biol Biotechnol* **3**: 294–299.

Grafen A (1989). The phylogenetic regression. *Phil Trans R Soc B* **326**: 119-157.

Hebert PDN, Cywinska A, Ball SL, DeWaard JR (2003). Biological identifications through DNA barcodes. *Proc R Soc B Biol Sci* **270**: 313–321.

Nazari V, Zakharov EV, Sperling FAH (2007). Phylogeny, historical biogeography, and taxonomic ranking of Parassiinae (Lepidoptera, Papilionidae) based on morphology and seven genes. *Mol Phylogenet Evol* **42**: 131-156.

Pagel M (1999). Inferring the historical patterns of biological evolution. *Nature* **401**: 877-884.

Paradis E, Claude J, Strimmer K (2004). APE: analyses of phylogenetics and evolution in R language. *Bioinformatics* **20**: 289–290. R package version 4.1.

Prum RO, Berv JS, Dornburg A, Field DJ, Townsend JP, Lemmon EM, Lemmon AR (2015). A comprehensive phylogeny of birds (Aves) using targeted next-generation DNA sequencing. *Nature* **526**: 569-573.

Robert J. Hijmans (2017). raster: Geographic Data Analysis and Modeling. R package version 2.6-7. https://CRAN.R-project.org/package=raster

Ratnasingham S, Hebert PDN (2013). A DNA-Based Registry for All Animal Species: The Barcode Index Number (BIN) System. *PLoS One* **8**: e66213.

Revell LJ. (2017). phytools: Phylogenetic tools for comparative biology (and other things). R package version 0.6-20.

Sanderson MJ (2002). Estimating absolute rates of molecular evolution and divergence times: a penalized likelihood approach. *Mol Biol Evol* **19**: 101-109.

Schliep KP (2011). phangorn: phylogenetic analysis in R. *Bioinformatics* **27**: 592–593. R package version 2.4.0.

Stamatakis A (2014). RAxML version 8: A tool for phylogenetic analysis and post-analysis of large phylogenies. *Bioinformatics* **30**: 1312-1313.

Swofford DL (2002). PAUP*: Phylogenetic Analysis Using Parsimony (*and other methods). Sunderland, MA.

Tamura K, Nei M (1993). Estimation of the number of nucleotide substitutions in the control region of mitochondrial DNA in humans and chimpanzees. *Mol Biol Evol* **10**: 512–526.

Welch JJ, Waxman D (2008). Calculating independent contrasts for the comparative study of substitution rates. *J Theor Biol* **251**: 667–678.

**Fig. S1.**

Sister lineages discovered for A) Annelida (*n* =65), B) Actinopterygii (*n* =435), C) Arachnida (*n* =366), D) Cnidaria (*n*=18), E) Coleoptera (*n*=844), F) Collembola (*n*=64), G) Diptera within Australasia (*n*=50), H) Diptera within Eurasia+Africa (*n*=419), I) Diptera within North America (*n*=929), J) Diptera within South America (*n*=154), K) Hymenoptera AUS (*n*=58), L) Hymenoptera EUR+AFR (*n*=419), M) Hymenoptera SA (*n*=92), N) Remaining Insect Orders (*n*=811), O) Lepidoptera AUS (*n*=356), P) Lepidoptera EUR+AFR (*n*=1042), Q) Lepidoptera NA (*n*=753), R) Lepidoptera SA (*n*=502), S) Malacostraca (*n*=111), T) Mollusca (*n*=91). All pairings were separated by a minimum of 20 degrees in median absolute latitude and 0.02-0.15 sequence divergence. The point for each BIN included in a pair is plotted according to its median latitude and median longitude on Kavrayskiy VII map projections using the data visualization software plotly (<https://plot.ly/>).

**A**

**B**

**C**

**D**

**E**

**F**

**G**

**H**

**I**

**J**

**K**

**L**

**M**

**N**

**O**

**P**

**Q**

**R**

**S**

**T**
